# Genetic analysis reveals candidate species in the *Scinax catharinae* clade (Amphibia: Anura) from Central Brazil

**DOI:** 10.1590/1678-4685-GMB-2015-0037

**Published:** 2016

**Authors:** Lídia Nogueira, Mirco Solé, Sérgio Siqueira, Paulo Roberto Antunes de Mello Affonso, Christine Strüssmann, Iracilda Sampaio

**Affiliations:** 1 Instituto Federal de Educação, Ciência e Tecnologia, Valença, BA, Brazil; 2 Departamento de Ciências Biológicas, Universidade Estadual de Santa Cruz, Ilhéus, BA, Brazil; 3 Departamento de Ciências Biológicas, Universidade Estadual do Sudoeste da Bahia, Jequié, BA, Brazil; 4 Departamento de Ciências Básicas e Produção Animal, Universidade Federal de Mato Grosso, Cuiabá, MT, Brazil; 5 Instituto de Estudos Costeiros, Universidade Federal do Pará, Bragança, PA, Brazil

**Keywords:** Amphibians, DNA barcode, Cerrado, rDNA

## Abstract

*Scinax* (Anura: Hylidae) is a species-rich genus of amphibians (113 spp.), divided into five species groups by morphological features. Cladistic analyses however revealed only two monophyletic clades in these groups: *Scinax catharinae* and *Scinax ruber*. Most species from the *S. catharinae* clade are found in Atlantic rainforest, except for *Scinax canastrensis,S. centralis, S. luizotavioi, S. machadoi,S. pombali* and *S. skaios*. In the present work, specimens of *Scinax* collected in Chapada dos Guimarães, central Brazil, were morphologically compatible with species from the*S. catharinae* group. On the other hand, genetic analysis based on mitochondrial (16S and 12S) and nuclear (rhodopsin) sequences revealed a nucleotide divergence of 6 to 20% between *Scinax* sp. and other congeners from the Brazilian savannah (Cerrado). Accordingly, Bayesian inference placed *Scinax* sp. in the *S. catharinae* clade with high support values. Hence, these findings strongly indicate the presence of a new species in the *S. catharinae* clade from the southwestern portion of the Brazilian savannah. To be properly validated as a novel species, detailed comparative morphological and bioacustic studies with other taxa from Brazil such as*S. canastrensis, S. centralis, S. luizotavioi, S. machadoi, S. pombali* and *S. skaios* are required.

The genus *Scinax* encompasses 113 species with a widespread distribution from southern Mexico to Argentina, Uruguay, St. Lucia and Trinidad and Tobago islands (Frost, 2015). Duellman and Wiens (1992) recognized seven species groups in this genus by means of morphological analyses (S. *catharinae, S. perpusillus, S. rizibilis, S. rostratus, S. ruber, S. staufferi* and *S. x-signatus*). Later, the groups S. *rizibilis* and *S. x-signatus* were regarded as synonyms of S. *catharinae* and S. ruber, respectively (Pombal *et al.*, 1995a). Cladistic inferences however recovered only two monophyletic clades: *S. catharinae* (including the groups *S. catharinae, S. staufferi* and *S. perpusillus*) and *S. ruber* (encompassing the groups *S. rostratus, S. ruber* and some species within *S. staufferi*) (Faivovich, 2002; Faivovich *et al.*, 2005).

The *S. catharinae* group (Frost, 2015) is characterized by the lack of an anterior process in the suprascapula, *m. depressor mandibulae* without an origin at the dorsal fascia of the *m. dorsalis scapulae*, distal division of the middle branch of the *m. extensor digitorum comunis longus*, and insertion of this muscle at the medial side on the tendon of the *m. extensor brevis medius digiti IV* (Faivovich, 2002). The vocalization of frogs from this group is usually composed of short notes and, sometimes, displays harmonic structure (Pombal *et al.*, 1995a, b).

Most species in this group are distributed throughout the Atlantic rainforest (Faivovich *et al.*, 2005). The only exceptions reported so far include *S. canastrensis, S. centralis, S. luizotavioi, S. machadoi, S. pombali* and *S. skaios*, which were observed in gallery forests within the Brazilian savannah (Cerrado) and in central and southeastern Brazil (Pombal and Bastos, 1996;Pombal *et al.*, 2010; Lourenço *et al.*, 2013).

During inventories of herpetofauna carried out for the Management Plan of Chapada dos Guimarães National Park in the southwestern Cerrado, some samples of*Scinax* morphologically compatible with species of *S. catharinae* group were collected, but these specimens were differentiated from all other species described so far. Therefore, the goal of the present study was to perform a molecular analysis of these samples as an additional tool to their taxonomic identification, besides verifying the presence of a putative new representative in the*S. catharinae* clade in areas distant from their center of origin.

Eight individuals of *Scinax* sp. were collected on April 04, 2006 in deep gallery forests alongside headwaters of the Coxipó River, in Chapada dos Guimarães, state of Mato Grosso, Brazil (Figure 1, Table 1). The specimens were deposited in the Vertebrate Collection of the Universidade Federal de Mato Grosso (UFMT). Approximately 25 mg of muscle were removed from the inner thigh of each specimen and preserved in ethanol 95% at – 20 °C for molecular analyses.

**Figure 1:**
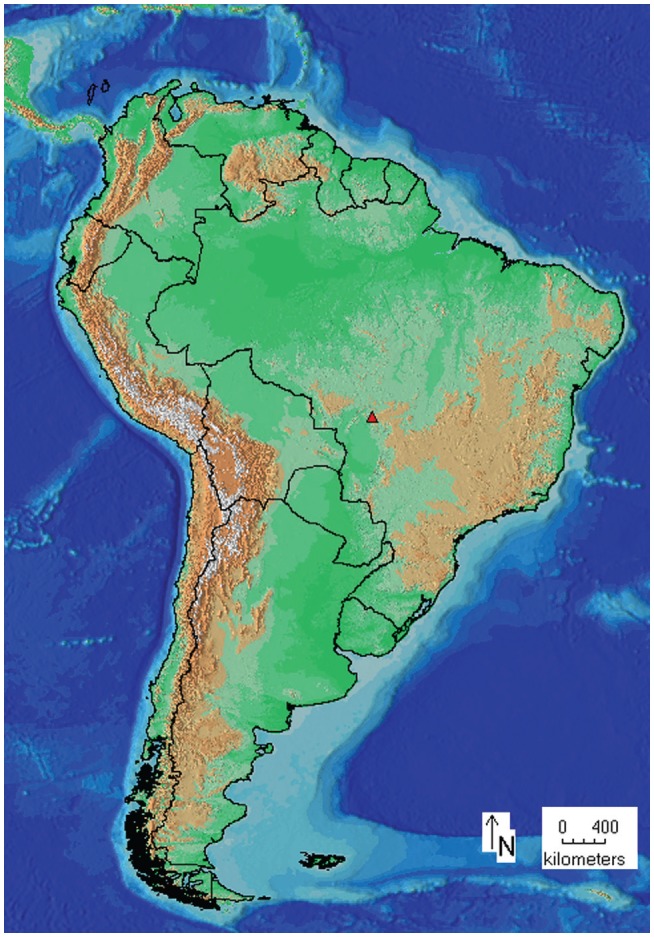
Map of Brazil showing the collection sites of *Scinax* sp. in Chapada dos Guimarães, Mato Grosso, Brazil (red triangle).

**Table 1:** Description of anuran samples used in the present study.

Voucher	Species	Clade	Country/Locality/State	Coordinates	GenBank Accession Number		
					16S	12S	Rhodopsin
MACN 36999	*Hypsiboas faber*		Argentina: San Vicente, Misiones	26°37'S54°08'W	AY549333	AY549333	AY844607
MZUESC9759	*Scinax agilis*	*S. catharinae*	Brazil: Conceição da Barra, Espirito Santo	18°25'S, 39°42'W	KT438894	KT438883	KT438902
–	*Scinax berthae*	*S. catharinae*	Argentina: Buenos Aires	34°36'S 58°22'W	AY843754	AY843754	AY844740
MCP3734	*Scinax catharinae*	*S. catharinae*	Brazil: São Francisco de Paula, Rio Grande Do Sul	29°26'S 50°35'W	AY843756	AY843756	AY844742
MVZFC 14457	*Scinax elaeochroa*	*S. ruber*	Costa Rica: Heredia	10°07'N83°33'W	AY843757	AY843757	AY844743
WED 54071	*Scinax garbei*	*S. ruber*	Ecuador: Riobamba, Chimborazo	01°40'S78°38'W	AY326033	AY326033	DQ283759
MACN 38650	*Scinax nasicus*	*S. ruber*	Argentina: Buenos Aires	34°36'S 58°22'W	AY843759	AY843759	AY844745
LH401	*Scinax* sp.	*S. catharinae*	Brazil Chapada dos Guimarães, Mato Grosso	15°28' S, 55°48'W	KT438886	KT438875	KT43889
LH905	*Scinax* sp.	*S. catharinae*	Brazil Chapada dos Guimarães, Mato Grosso	15°28' S, 55°48'W	KT438887	KT438876	KT438898
LH900	*Scinax* sp.	*S. catharinae*	Brazil Chapada dos Guimarães, Mato Grosso	15°28' S, 55°48'W	KT438888	KT438877	KT438899
LH902	*Scinax* sp.	*S. catharinae*	Brazil Chapada dos Guimarães, Mato Grosso	15°28' S, 55°48'W	KT438889	KT438878	KT438900
LH908	*Scinax* sp.	*S. catharinae*	Brazil Chapada dos Guimarães, Mato Grosso	15°28' S, 55°48'W	KT438890	KT438879	KT438901
LH909	*Scinax* sp.	*S. catharinae*	Brazil Chapada dos Guimarães, Mato Grosso	15°28' S, 55°48'W	KT438891	KT438880	–
LH904	*Scinax* sp.	*S. catharinae*	Brazil Chapada dos Guimarães, Mato Grosso	15°28' S, 55°48'W	KT438892	KT438881	–
LH903	*Scinax* sp.	*S. catharinae*	Brazil Chapada dos Guimarães, Mato Grosso	15°28' S, 55°48'W	KT438893	KT438882	–
MZUESC11079	*Scinax strigilatus*	*S. catharinae*	Brazil Camacan, Bahia	15°24'S, 39°30'W	KT438895	KT438884	–
MZUESC11080	*Scinax strigilatus*	*S. catharinae*	Brazil Camacan, Bahia	15°24'S, 39°30'W	KT438896	KT438885	–
CFBH 5788	*Scinax uruguayas*	*S. ruber*	Brazil Cambará do Sul, Rio Grande do Sul	29°02'S, 50°08'W	AY843681	AY843681	AY844674

Total DNA was extracted by using the Wizard® Genomic Purification kit (Promega), following manufacturer's instructions. The primer pairs used to amplify 16S, 12S, and rhodopsin, respectively, were: L1- 5'GCCTCGC TTGTTTACCAAAAAC −3 (Palumbi, 1996) and H1 – 5'CCGGTCTGAACTCAGATCACGT 3' (Varela *et al.*, 2007); L1- 5'AAAAAGCTTCAAACTGGGATTAGAT ACCCCACTAT3' and H1- 5'TGACTGCAGAGGGTGA CGGGCGGTGTGT3' (Kocher *et al.*, 1989), and Rhod-L1 5'ACCATGAACGGAACAGAAGGYCC 3' and Rhod-H1 5'GTAGCGAAGAARCTTCAAMGTA 3' (Bossuyt and Milinkovitch, 2000).

The PCR conditions consisted of an initial denaturation step at 95 °C for 5 min, followed by 35 cycles of denaturation at 94 °C for 40 s, annealing at 55 °C (12S and 16S) or 49 °C (rhodopsin) for 40 s and extension at 72 °C for 30 s, plus a final extension step at 72 °C for 7 min. Subsequently, the reaction products were purified and sequenced in an ABI 3500XL Genetic Analyzer automatic sequencer (Applied Biosystems). Sequencing reactions were carried out by using terminal dideoxynucleotides (Sanger *et al.*, 1977). The sequences were then aligned with Clustal W available in the software BioEdit v. 5.09 (Hall, 1999). The software GBlocks 0.91 (Castresana, 2000) was used to eliminate poorly aligned positions and divergent region portions of 16S, according to the following parameters: minimum number of sequences for a flank position to 10, maximum number of contiguous nonconserved positions to 08, minimum length of a block to 2, and allowed gap positions to within half.

To estimate the divergence matrix and phylogeny we added sequences of seven other anuran species from GenBank to our data set: *S. catharinae, Scinax berthae, Scinax uruguayus, Scinax garbei, Scinax elaechroa, Scinax nasicus* and*Hypsiboas faber* (outgroup). Two other species from the *S. catharinae* clade collected in Bahia, northeastern Brazil and Espirito Santo, southeastern Brazil, were also included in our analysis: *Scinax strigilatus* and *Scinax agilis* (Table 1).

Genetic divergence was estimated using the Kimura-2-parameter (K2P) substitution model (Kimura, 1980) in the software MEGA v. 5.0 (Tamura *et al.*, 2011). The 16S, 12S and rhodopsin sequences were concatenated in the software DnaSP, v. 4.0 (Librado and Rozas, 2009).

A Bayesian phylogeny was inferred using the software MrBayes 3.1 (Ronquist and Huelsenbeck, 2003). The best mutation model was estimated according to Akaike Information Criteria – AIC in the software jModel Test 0.1 (Posada, 2008). Two runs (four chains each) with 20 million generations were performed with trees being sampled at every 1000 generations. Adequate burn-in was determined by examining likelihood scores of the heated chains for convergence on stationarity, as well as the effective sample size of values in Tracer 1.5 (Rambaut and Drummond, 2007). We discarded 10% of the generations/trees. We considered relationships strongly supported when posterior probabilities were equal to or higher than 0.95.

Eighteen sequences of 16S and 12S were obtained from each of the nine*Scinax* species, comprising 423 bp (164 variable sites) and 386 bp (131 variable sites) for each fragment respectively. For rhodopsin, 13 sequences of 316 bp with 51 variable sites were obtained from eight *Scinax*representatives.

The intraspecific nucleotide divergence in *Scinax* sp. was 0.2% for 16S, 0.3% for 12S, 0.2% for combined 12S+16S, and 0% for the rhodopsin. The nucleotide divergence of *Scinax* sp. in relation to the other species ranged from 6 to 13%, 7 to 20%, 6 to 18% and 0.6 to 6% for 16S, 12S, 16S+12S and rhodopsin, respectively (Table 2).

**Table 2:** Interspecific nucleotide divergence within *Scinax* (Anura: Hylidae) based on K2P model of 16S (above diagonal), combined 12S+16S (above diagonal in parentheses), 12S (below diagonal) and Rhodopsin (below diagonal in parentheses) genes. The species 1 to 5 belong to the *S. catharinae* clade, while the species 6 to 9 belong to the *S. ruber* clade; *H. faber* (10) was used as outgroup.

	Species	1	2	3	4	5	6	7	8	9	10
*S. catharinae* clade	1. *S. agilis*	–	0.10	0.11	0.10	0.10	0.12	0.13	0.19	0.18	0.16
			(0.05)	(0.06)	(0.06)		(0.06)	(0.03)	(0.05)	(0.04)	(0.05)
	2. *S. berthae*	0.10	–	0.05	0.06	0.05	0.10	0.12	0.17	0.15	0.15
		(0.10)		(0.03)	(0.03)		(0.06)	(0.04)	(0.04)	(0.06)	(0.06)
	3. *S. catharinae*	0.10	0.04	–	0.05	0.06	0.10	0.12	0.18	0.14	0.15
		(0.10)	(0.04)		(0.01)		(0.06)	(0.05)	(0.06)	(0.07)	(0.05)
	4. *Scinax* sp.	0.10	0.06	0.06	–	0.07	0.12	0.15	0.21	0.16	0.15
		(0.10)	(0.06)	(0.05)			(0.05)	(0.04)	(0.05)	(0.06)	(0.04)
	5. *S. strigilatus*	0.10	0.06	0.06	0.07	–	0.10	0.13	0.17	0.15	0.16
		(0.10)	(0.06)	(0.06)	(0.07)						
*S. ruber* clade	6. *S. uruguayus*	0.15	0.13	0.12	0.11	0.12	–	0.10	0.17	0.13	0.15
		(0.13)	(0.12)	(0.11)	(0.11)	(0.11)		(0.02)	(0.03)	(0.04)	(0.05)
	7. *S. elaeochrous*	0.13	0.13	0.13	0.13	0.12	0.11	–	0.16	0.12	0.15
		(0.13)	(0.12)	(0.12)	(0.13)	(0.12)	(0.10)		(0.01)	(0.02)	(0.04)
	8. *S. nasicus*	0.12	0.13	0.13	0.13	0.12	0.14	0.07	–	0.16	0.21
		(0.15)	(0.14)	(0.15)	(0.16)	(0.14)	(0.15)	(0.11)		(0.03)	(0.06)
	9. *S. garbei*	0.14	0.13	0.14	0.12	0.13	0.11	0.10	0.13	–	0.20
		(0.15)	(0.14)	(0.14)	(0.13)	(0.14)	(0.11)	(0.10)	(0.14)		(0.05)
	10. Outgroup	0.16	0.15	0.15	0.15	0.16	0.17	0.14	0.16	0.15	–
		(0.16)	(0.15)	(0.15)	(0.15)	(0.16)	(0.16)	(0.15)	(0.18)	(0.17)	

The Bayesian consensus phylogeny (16S + 12S + rhodopsin) placed *Scinax*sp. as a distinct clade with strong support, being closely related to *S. berthae, S. catharinae, S. strigilatus* and *S. agilis*, all belonging to the *S. catharinae* clade (Figure 2 and Table 2). The four species from the *S. ruber* clade also formed a monophyletic group with strong support.

**Figure 2:**
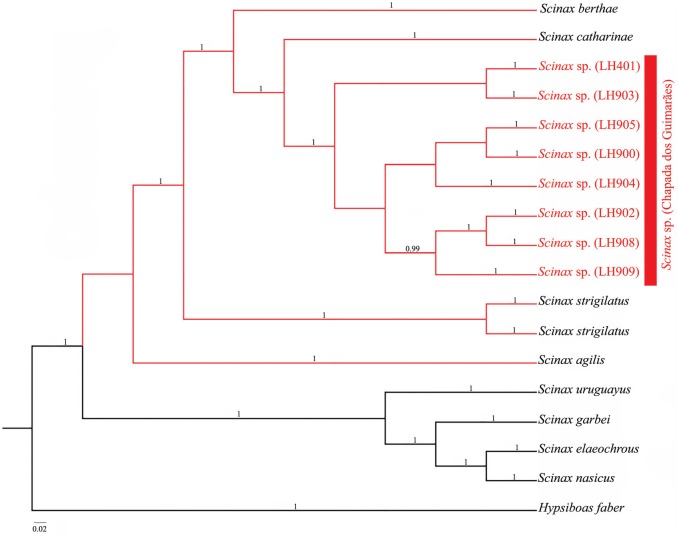
Bayesian consensus phylogeny based on combined analysis of 12S, 16S and rhodopsin (1,123 bp) of *Scinax* species, using *H. faber* as outgroup. Posterior probabilities higher than 0.95 are shown. The *S. catharinae* clade is highlighted in red while the*S. ruber* clade is highlighted in black. *Scinax sp.* (Chapada dos Guimarães, Mato Grosso, Brazil) corresponds to the specimens collected in this study.

Even though the cytochrome C oxidase I (COI) gene has been elected as a universal DNA barcode in animals (Hebert *et al.*, 2003), the 16S gene seems to be more effective to discriminate amphibian species (Vences *et al.*, 2005), thus being used in the present study. Indeed, the genetic distances of 7 to 10% in 16S rDNA observed between *Scinax* sp. and the other known species in the*S. catharinae* clade (*S. berthae, S. catharinae, S. strigilatus* and *S. agilis*) (Table 2) are higher than the minimum value of 3% in nucleotide divergence proposed by Fouquet *et al.*(2007) to discriminate anuran species. Moreover, sequences of 12S and rhodopsin (nuclear) were also included to provide additional support to our hypothesis of a new species in the *S. catharinae* clade occuring in the Chapada dos Guimarães.

Many researchers advocate the integration of multiple approaches (molecular, cytogenetic, morphological and ecological studies) for identifying species (Dayrat, 2005; Padial *et al.*, 2010). According to the nomenclature rules established byVieites *et al.* (2009),*Scinax* sp. could be classified as an "unconfirmed candidate species" (UCS), depending on additional morphological, ecological and vocalization studies to confirm its taxonomic status.

In conclusion, our molecular data provide evidence of a new species in the *S. catharinae* clade occurring in the Chapada dos Guimarães region, central Brazil. However, further morphological and bioacoustical analyses should be performed and focused on comparative data with other species from the *S. catharine* clade from Brazilian savannah, such as *S. canastrensis, S. centralis, S. luizotavioi, S. machadoi, S. pombali* and *S. skaios*.
